# Fast and Reliable PCR Amplification from *Aspergillus fumigatus* Spore Suspension Without Traditional DNA Extraction

**DOI:** 10.1002/cpmc.89

**Published:** 2019-08-20

**Authors:** Marcin G. Fraczek, Can Zhao, Lauren Dineen, Ressa Lebedinec, Paul Bowyer, Michael Bromley, Daniela Delneri

**Affiliations:** ^1^ Manchester Institute of Biotechnology, Division of Evolution & Genomic Sciences, School of Biological Sciences, Faculty of Biology Medicine and Health University of Manchester Manchester United Kingdom; ^2^ Manchester Fungal Infection Group, Core Technology Facility, Manchester, Division of Infection, Immunity and Respiratory Medicine, School of Biological Sciences, Faculty of Biology, Medicine and Health University of Manchester Manchester United Kingdom

**Keywords:** *Aspergillus fumigatus*, DNA extraction, filamentous fungi, spore PCR

## Abstract

*Aspergillus fumigatus* is an opportunistic human pathogenic mold. DNA extraction from this fungus is usually performed by mechanical perturbation of cells, as it possesses a rigid and complex cell wall. While this is not problematic for single isolates, it can be time consuming for large numbers of strains if using traditional DNA extraction procedures. Therefore, in this article we describe a fast and efficient thermal‐shock method to release DNA from spores of *A. fumigatus* and other filamentous fungi without the need for complex extraction methods. This is especially important for high‐throughput PCR analyses of mutants in 96‐ or 384‐well formats in a very short period of time without any concern about sample cross‐contamination. This method is currently being used to validate the protein‐coding gene and non‐coding RNA knockout libraries in *A. fumigatus* generated in our laboratory, and could be used in the future for diagnostics purposes. © 2019 The Authors.

## INTRODUCTION

*Aspergillus fumigatus* is a filamentous fungus that causes severe allergy and life‐threating infections, especially in susceptible and immunocompromised patients (Macdonald et al., 2019). The morbidity and mortality in such patients remain high (Brown et al., 2012), and therefore this mold is extensively studied in laboratories all over the world. Molecular methods to study the genetics of fungi often require PCR steps that use genomic DNA as a template. Traditionally, genomic DNA has been obtained from the cells by mechanical means such as grinding in a mortar and pestle, glass bead beating (Fraczek et al., 2013), or using expensive commercial DNA extraction kits. The challenge is to break the rigid and complex cell wall to release DNA from the cells (Gow, Latge, & Munro, 2017). Although usually effective, the fungal DNA extraction methods are time consuming and not designed for high‐throughput sample processing. Moreover, a number of these methods require a time‐consuming pre‐culturing step, as it is perceived that DNA is more readily isolated from fungal hyphae rather than spores.

To overcome these obstacles, we have developed a simple, fast, and efficient method to generate PCR products directly from *A. fumigatus* spores, as well as other filamentous fungi, without the need to use the time‐consuming and usually expensive DNA extraction kits. This is particularly useful for gene knockout strain confirmation or simple gene amplification for Sanger sequencing and other applications. Our technique can not only be applied in single‐tube format but also in high‐throughput plates (i.e., 96‐ or 384‐well) without any concern about sample cross‐contamination. This method does not require specialized training, expensive equipment, or complex sample preparation, and DNA from 96/384 samples can be obtained in less than 30 min. Currently, this methodology is being used to validate protein‐coding gene and non‐coding RNA knockout *A. fumigatus* libraries generated in our laboratory, and it could be used in the future for diagnostics purposes.

*CAUTION: A. fumigatus* is a Biosafety Level 2 (BSL‐2) organism. Appropriate guidelines and regulation must be followed when handling this organism or any other pathogenic mold. All work with fungal spores must be carried out in a Biosafety Class II Cabinet and all consumables and fungal spores must be disposed of according to local guidelines.

## Basic Protocol 1 PREPARATION OF SPORE SUSPENSION AS A PCR TEMPLATE

This protocol describes preparation of a fungal spore suspension for PCR. If spores are already harvested, the user should proceed to Basic Protocol 2.

*A. fumigatus* spores are obtained from solid agar cultures grown on Sabouraud agar (SAB) at 37°C for 2 to 3 days. Growth conditions for other fungal species may vary. Subsequently, spores are harvested in PBS–Tween 20 solution (with or without 20% [v/v] glycerol) and at this stage they are ready to be used as a PCR template or can be stored at 4°C.

### Materials

SAB agar (see recipe) or fungal species–specific growth medium
Frozen stock of fungal spores
PBS–Tween 20 solution (see recipe)

25‐cm^2^ vented tissue culture flasks (growth flasks; e.g. Corning, 430639)
Plastic, disposable inoculation loops
37°C incubator (for *A. fumigatus*; temperature may vary for other fungal species)
Miracloth (Merck) or equivalent sterile filtration material
15‐ or 50‐ml conical tube (e.g., Corning Falcon) for spore suspension

Additional reagents and equipment for counting using hemocytomter (see Current Protocols article: Stevenson, 2006)

1 Pour SAB agar into 25‐cm^2^ vented tissue culture flasks to cover the surface and let it set.
2 Defrost fungal spores and spread them on top of SAB agar in the tissue culture flasks using a plastic, disposable loop. Incubate for 2 to 3 days at 37°C until a sufficient amount of spores are observed.
  For A. fumigatus, the most common solid growth media used are SAB agar and Aspergillus complete agar, and optimal incubation temperature is 37°C. The growth medium and temperature may vary between fungal species, and specific conditions should be used for these species to generate spores. Some fungal species may also require light for spore formation.
  Culturing filamentous fungi in vented tissue culture flasks as opposed to Petri dishes significantly reduces contamination and risk of spore dispersion in the air.
3 Harvest the spores by pouring approximately 10 ml of 1× PBS–Tween 20 (with or without 20% [v/v] glycerol) solution into the growth flasks; mix well.
  Tween 20 is a nonionic detergent/dispersing agent commonly used in spore preparation (alternatively, Tween 80 can be used). It makes the dispersion of hydrophobic spores in PBS easier.
  The described spore PCR protocol also works well for spores suspended in PBS–Tween 20 solution containing 20% (v/v) glycerol. This solution is usually used to preserve the spores at −80°C.
4 Pour the PBS–Tween 20‐spores solution through a sterile filtration material such as Miracloth into a 15‐ or 50‐ml conical tube.
5 Optional: Count the spores using a hemocytometer or any other cell counter (see Current Protocols article: Stevenson, 2006).
  If large numbers of strains are being tested, as for high‐throughput experiments, spore counting may be problematic and can be omitted. In this case, we recommend continuing with the protocol, as in our hands PCR amplification has worked from spore supernatants from as few as 7.8 × 10^5^/ml and as many as 8 × 10^8^/ml A. fumigatus spores.
  We have not tested the spore template range (i.e., lowest and the highest spore concentrations) for other fungal species.
6 At this stage, spores are ready for spore PCR (see Basic Protocol 2), or they can be stored at 4°C.

## Basic Protocol 2 DNA TEMPLATE PREPARATION AND SPORE PCR

Following spore harvest (see Basic Protocol 1), the spore suspension is subjected to extreme temperature treatment in order to make the genomic DNA accessible for PCR. No mechanical treatment is required.

### Materials

Spore suspension (Basic Protocol 1)
Primers (5 µM each, forward and reverse; experiment specific)
LongAmp Taq DNA polymerase with 5× reaction buffer (New England Biolabs; M0323)
dNTP mix (5 mM of each dNTP)
Sterile, molecular‐grade water
For agarose gel electrophoresis (also see Current Protocols article: Voytas, 2000):
  6× DNA loading dye
  1× TAE buffer (see recipe)
  Ethidium bromide or other DNA gel stain

0.2‐ml PCR tubes, 0.2‐ml/well 96‐well PCR plates, or 150 µl/well 384 well PCR plates with tight (preferably aluminum) seals
Thermal cycler
Centrifuge
UV transilluminator

Additional reagents and equipment for agarose gel electrophoresis (see Current Protocols article: Voytas, 2000)

1 Transfer 30 µl of previously prepared spore suspension (see Basic Protocol 1) to 0.2‐ml PCR tubes or PCR plates and seal tightly (preferably using aluminum seals).
  The spore suspension should be mixed thoroughly before transferring to the tubes or plates.
2 Incubate the tubes or plates with the spore suspension at 95°C for 15 min in a thermal cycler.
  A water bath or 95°C incubator can also be used.
3 Immediately transfer the tubes/plates to an −80°C freezer and leave for approximately 10 min (to perform thermal shock).
  Steps 2 and 3 are critical for subsequent successful PCR amplification. We have noticed that a better PCR yield is obtained if the spore suspension is immediately transferred to −80°C from 95°C.
4 Defrost the spore suspension at room temperature.
5 Centrifuge for 1 min at maximum speed to remove cellular debris and keep the supernatant for PCR.
  Following the thermal shock (steps 2 and 3), the spore suspension can also be used directly as PCR template, omitting the centrifugation step; however, we observed a slightly better PCR yield if the spores are centrifuged and only the supernatant is used for PCR. For some fungal species, centrifugation of spores may be necessary to avoid transferring cell debris into the PCR reaction and to prevent inhibition during PCR reaction.
6 Prepare the following PCR mixture in PCR tubes or PCR plates. The following volumes are per one reaction:
  1 to 3 µl of thermally treated spore suspension supernatant
  1 µl of 5 µM forward primer
  1 µl of 5 µM reverse primer
  5 µl of 5× LongAmp Taq DNA reaction buffer
  0.5 µl of 2500 U/ml LongAmp Taq DNA polymerase
  0.5 µl of dNTP mix
  Make up to 25 µl with sterile, molecular‐grade water.
7 Place the tubes/plates in a thermal cycler and use the following PCR conditions for the LongAmp Taq DNA polymerase:

**Table:** 

1 cycle:	1 min	95°C
35 cycles:	20 s	95°C
	20 s	55°‐60°C
	50 s/kb expected PCR product	65°C
1 cycle:	5 min	65°C
  For most ∼20‐bp primers, the annealing temperature between 55°C and 60°C is sufficient to amplify a PCR product using the LongAmp Taq DNA polymerase and the conditions specified.
  Successful PCR amplification has also been obtained with a Phusion High‐Fidelity DNA polymerase (New England Biolabs; M0530; see Fig. 1A). This polymerase was tested in the following PCR conditions for PCR products of ∼1.2 kb: 1 cycle at 98°C for 30 s followed by 35 cycles of 98°C for 10 s, 58°C for 20 s, 72°C for 45 s, and finally 1 cycle of 72°C for 5 min.

**Figure 1:**
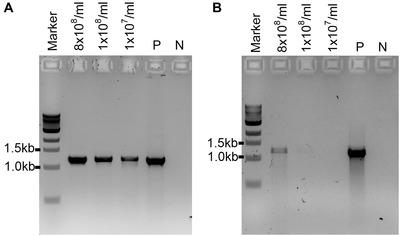
PCR to test the efficiency of polymerases in amplifying PCR products from supernatants from different spore concentrations of the *A. fumigatus* wild‐type strain with primers ITS1/D2 (expected PCR band sizes is ∼1.2 kb). (**A**) Phusion High‐Fidelity DNA polymerase (New England Biolabs). (**B**) MyTaq RED Mix DNA polymerase (Bioline). P: positive PCR control amplified from genomic DNA (50 ng) of the *A. fumigatus* wild‐type strain; N: negative control (no DNA).

8 Following the PCR, mix 5 µl of the PCR reaction with 1 µl of 6× DNA loading dye and load the reactions on an agarose gel (Voytas, 2000).
  The gel concentration depends on the size of the amplified product. For gels between 600 bp and 1.5 kb, we use a 1.5% agarose gel.
9 Analyze the stained gel under the UV light in a UV transilluminator for the presence/absence of the expected‐size PCR products.

## REAGENTS AND SOLUTIONS

### PBS–Tween 20, 1×

Prepare in distilled water:
10 mM Na_2_HPO_4_
137 mM NaCl
1.8 mM KH_2_PO_4_
2.7 mM KCl
0.05% (v/v) Tween 20
20% (v/v) glycerol (optional—for spores to be stored at −80°C)
Adjust to pH 7.4 and autoclave
This solution can be stored at room temperature for several months

### SAB agar

Prepare in distilled water:
1% (w/v) bacteriological peptone
4% (w/v) glucose
2% (w/v) technical or biological agar
Adjust to pH 5.6 and autoclave. Cool to ∼55°C, pour into 25‐cm^2^ vented tissue culture flasks, and allow to set.
Store flasks with solidified agar up to several months at room temperature

## COMMENTARY

### Background Information

PCR is one of the most commonly used methods for analysis and genomic manipulation of filamentous fungi in a molecular laboratory setting. Its simplicity allows quick and efficient amplification of desired sequences from the genome or analysis of mutant strains. However, it requires genomic DNA obtained from fungal cells (spores or hyphae), which are surrounded by the rigid and complex cell wall structure. Most commonly, DNA is released from the cells by mechanical means such as use of glass beads or grinding in liquid nitrogen. Although high‐quality DNA is obtained from spores by glass‐bead beating (Fraczek et al., 2013), grinding in liquid nitrogen usually requires fungal cultures to be grown for several days in order to obtain hyphae. This becomes problematic especially if large numbers of mutants need to be analyzed. Therefore, in this article we describe a new and reliable high‐throughput method to amplify PCR products directly from spores of *A. fumigatus* as well as other filamentous fungi, without the need for hyphae generation or mechanical treatments. This methodology can be applied to analyze a great number of mutants simultaneously, which significantly reduces time and costs. Moreover, there is no concern about sample cross‐contamination or spore environmental exposure since there is little manipulation of the spore suspension.

**Figure 2:**
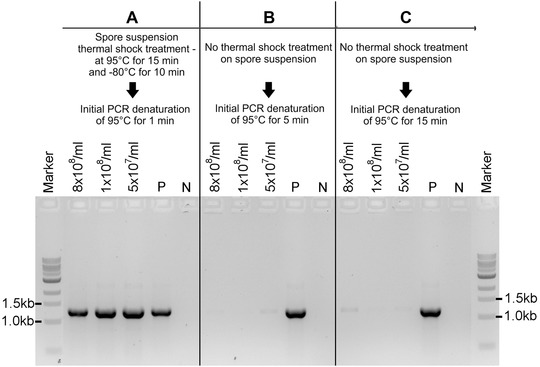
Thermal shock is crucial for successful PCR amplification from fungal spores. *A. fumigatus* spores at three different concentrations (8 × 10^8^/ml, 1 × 10^8^/ml, and 5 × 10^7^/ml) were prepared as described in Basic Protocol 1. 3 µl of spore suspension were used as the DNA template for PCR. (**A**) PCR result from spore suspension subjected to thermal shock of 95°C for 15 min and −80°C for 10 min prior to PCR, as described in Basic Protocol 2. No thermal shock was done for spore suspensions from Panels **B** and **C**. Subsequently, a PCR was run with an initial denaturation step of 95°C for 1 min (A), 5 min (B), or 15 min (C) with primers ITS1 and D2 (expected PCR product ∼1.2 kb) and LongAmp Taq DNA polymerase with PCR conditions described in Basic Protocol 2. P: positive PCR control amplified from genomic DNA (50 ng) of the *A. fumigatus* wild‐type strain; N: negative control (no DNA).

The described “spore PCR” protocol was developed to facilitate the analysis of *A. fumigatus* protein‐coding gene and non‐coding RNA knockout libraries created in our laboratory. First, we tested if application of a spore suspension as a DNA template for PCR could generate PCR products. Based on the *Saccharomyces cerevisiae* colony‐PCR methodology (Parker et al., 2017), we hypothesized that high temperature would be sufficient to release DNA from *Aspergillus* spores. To this end, we used an initial PCR denaturation temperature of 95°C for both 5 and 15 min with three different *A. fumigatus* spore concentrations (8 × 10^8^/ml, 1 × 10^8^/ml, and 5 × 10^7^/ml). Primers ITS1 (5′‐TCCGTAGGTGAACCTGCGG‐3′) and D2 (5′‐TTGGTCCGTGTTTCAAGACG‐3′) were used to target the fungal ribosomal DNA (rDNA) sequences. However, very low amounts of amplified products were observed after the PCR (Fig. 2B and 2C). Then, we treated the spore suspension for 15 min at 95°C immediately followed by 10 min at −80°C before the initial PCR denaturation of 95°C for 1 min. This step significantly improved PCR amplification (Fig. 2A), which shows that the thermal shock from 95°C to −80°C is crucial for the DNA release from fungal spores.

**Figure 3:**
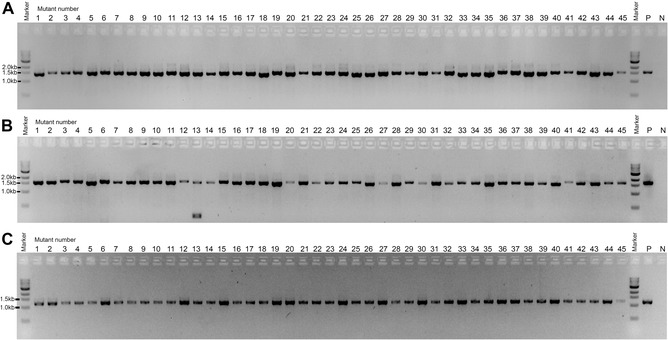
To verify the correct integration of the hygromycin B phosphotransferase *(hph*) marker in 45 different *A. fumigatus* putative gene knockout mutants, spore PCR was carried out with the primer pairs P1/hphsqR2 (**A**; amplification of the DNA spanning the upstream flanking region of the gene of interest and *hph*) and P4/hphsqF2 (**B**; amplification of the DNA spanning between the *hph* and the downstream flanking region of the gene of interest). Primers P1 and P4 are complementary to the upstream and downstream flanking regions of the gene of interest, respectively. Primers hphsqF2 and hphsqR2 target the sequence of the *hph* used to replace the genes of interest. The expected PCR band sizes for the correct knockout mutants are ∼1.5 kb. P: positive PCR control amplified from genomic DNA (50 ng) of a known *A. fumigatus* knockout strain; N: negative control (no DNA). (**C**) Spore PCR on the supernatant of the 45 *A. fumigatus* gene knockout mutants validated in Panels A and B using primer pair ITS1/D2. This PCR confirms the amplification of fungal rDNA and that the size of the correct amplification product is ∼1.2 kb. P: positive PCR control amplified from genomic DNA (50 ng) of the *A. fumigatus* wild‐type strain; N: negative control (no DNA).

Subsequently, we successfully confirmed 45 different *A. fumigatus* protein gene knockout mutants using this methodology with primer sets P1/hphsqR and P4/hphsqF, each generating PCR products of approximately 1.5 kb (Fig. 3A and 3B). Primers hphsqR (5′‐CACCGGTCAACCATGATCTG‐3′) and hphsqF (5′‐CCCAGCACTCGTCCGAGGGC‐3′) target the marker gene hygromycin B phosphotransferase (*hph*), which was used to replace the gene of interest [see Current Protocols article Zhao et al. (2019) for detailed protocol]. Primers P1 and P4 are specific for the upstream and downstream flank sequences of the deleted gene, respectively. As a PCR control, we used the same spore supernatant with primers ITS1 and D2, generating PCR products of approximately 1.2 kb (Fig. 3C).

**Figure 4:**
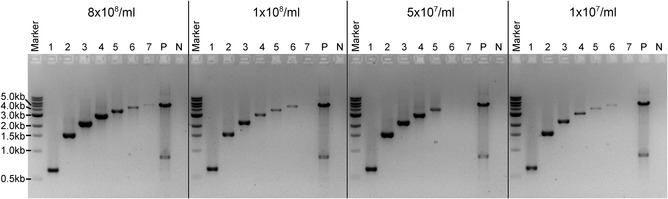
Spore PCR of the *AYG1* gene and its flanking regions to test the range of PCR products effectively amplified using different concentrations of *A. fumigatus* wild‐type spores. The expected PCR band sizes are as follows: 622 bp (1), 1546 bp (2), 2241 bp (3), 3005 bp (4), 3744 bp (5), 4515 bp (6), and 5259 bp (7); P: positive control for the PCR reaction using genomic DNA (50 ng) of the *A. fumigatus* wild‐type strain as template, with the expected band size of 5259 bp; N: negative control (no DNA).

Then, a number of PCR products of increasing sizes were amplified to test the size range of the PCR amplification using this methodology (Fig. 4). The PCR products ranged from 622 to 5259 bp (7 amplicons in total) and amplified the *A. fumigatus AYG1* gene (Afu2g17550) and its flanking regions using the supernatant from four different spore concentrations (8 × 10^8^/ml, 1 × 10^8^/ml, 5 × 10^7^/ml, and 1 × 10^7^/ml). Using the supernatant of the highest spore concentration as template (8 × 10^8^/ml), all the PCR products were amplified and were visible on an agarose gel up to 5.2 kb. The PCR product with the highest size to be amplified from all templates was ∼3.7 kb. In general, PCR products between ∼0.6 and ∼3.0 kb amplify better.

**Figure 5:**
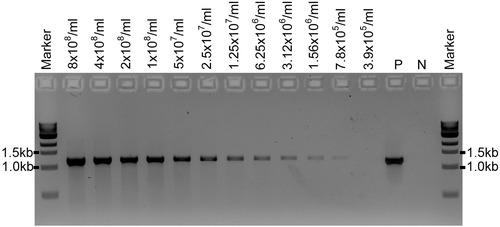
PCR amplification from a range of spore concentrations. 1 µl of the spore supernatant from different known concentrations of *A. fumigatus* spores was used in the PCR reaction with primers ITS1/D2 (expected PCR band sizes is ∼1.2 kb). The spore concentration in the spore suspension is indicated as number of spores per milliliter. 8 × 10^8^ spores/ml and 3.9 × 10^5^ spores/ml are, respectively, the highest and lowest concentrations tested. P: positive PCR control amplified from genomic DNA (50 ng) of the *A. fumigatus* wild‐type strain; N: negative control (no DNA).

We also tested the minimum and maximum spore concentrations needed to generate the PCR products (Fig. 5). Here, 1 µl of the supernatant from different known concentrations of *A. fumigatus* spores was used in the PCR reaction with primers ITS1 and D2. PCR amplicons were observed from the spore supernatant containing as few as 7.8 × 10^5^/ml and as many as 8 × 10^8^/ml spores.

LongAmp Taq DNA polymerase (New England Biolabs) was used in our experiments with great success. Two other polymerases, Phusion High‐Fidelity DNA polymerase (New England Biolabs; M0530) and MyTaq RED Mix DNA polymerase (Bioline; BIO‐25043), were also tested with the described protocol using the PCR conditions specified by the manufacturers (Fig. 1). The Phusion High‐Fidelity DNA polymerase was shown to amplify rDNA products from *A. fumigatus* spore supernatant with comparable PCR yield to the LongAmp Taq DNA polymerase (Fig. 1A). However, in our hands, amplification using the MyTaq RED Mix DNA polymerase has not produced sufficient amounts of PCR products (Fig. 1B).

**Figure 6:**
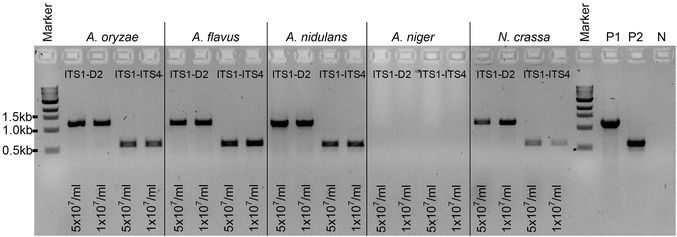
Spore PCR using the supernatant from spore suspensions of different filamentous fungi with primers ITS1/D2 (expected PCR band size is ∼1.2 kb) and ITS1/ITS4 (expected PCR band size ∼600 bp). Two different spore concentrations were tested (i.e., 5 × 10^7^/ml and 1 × 10^7^/ml). 1 µl of the supernatant was used in the PCR reaction with the LongAmp Taq DNA polymerase. Positive PCR controls were amplified from DNA (50 ng) of the *A. fumigatus* wild‐type strain with primers ITS1/D2 (P1) and ITS1/ITS4 (P2). N: negative control (no DNA).

**Figure 7:**
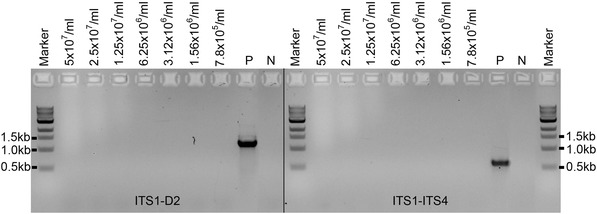
Spore PCR using the supernatant of a spore suspension of *A. niger* at different concentrations. Primers ITS1/D2 and ITS1/ITS4 were used. P: positive PCR control amplified from the *A. niger* genomic DNA (50 ng) wild‐type strain; N: negative control (no DNA).

The described spore PCR methodology was developed for the pathogenic fungus *A. fumigatus*; however, we have applied it with success to other molds such as *A. oryzae*, *A. flavus*, *A. nidulans*, and *Neurospora crassa*, as shown in Figure 6. Here, for each fungus, rDNA sequences were amplified with primer sets ITS1/D2 and ITS1/ITS4 (ITS4: 5′‐TCCTCCGCTTATTGATATGC‐3′) from supernatant taken from spore suspensions at two different concentrations (5 × 10^7^/ml and 1 × 10^7^/ml). The expected size of the PCR products is ∼1.2 kb for ITS1/D2 and ∼600 bp for ITS1/ITS4. The only mold from the 6 molds tested for which this protocol failed to amplify any rDNA PCR fragments is *A. niger* (Fig. 6). Further experiments on the *A. niger* spore supernatant using a wider range of spore concentrations (from 5 × 10^7^/ml to 7.8 × 10^5^/ml) also failed to amplify any PCR products (Fig. 7), suggesting that there may be a higher amount of PCR inhibitors in the spore supernatant of this fungus or the genomic DNA released from the spore suspension using this protocol is not in sufficient quantity.

We have not tested any other filamentous fungal species. Therefore, if a different mold species is being used, we recommend testing the above protocol with the primers targeting rDNA in the first instance.

### Critical Parameters and Troubleshooting

The thermal shock step is of key importance in this protocol. We showed that exposing the spore suspension to extreme temperatures, first at 95°C for 15 min, followed by −80°C for 10 min, is necessary for DNA release into the supernatant (Fig. 2A) and for PCR amplification of products with a size ranging between ∼600 bp and ∼5.3 kb. However, the recommended PCR amplification range is between ∼600 bp and ∼3 kb (Fig. 4). If no amplification is observed for the desired product, e.g., when testing a knockout strain for gene deletion, we suggest running a control PCR with primers amplifying an rDNA fragment (e.g., with primer pairs ITS1/ITS4 or ITS1/D2) to confirm that the DNA is released from the spores during the thermal shock treatment. If the rDNA fragment has been amplified, it is possible that the PCR for the desired amplicon failed because the size of the product is out of the recommended amplification range, there is a problem with the chosen primers, or the gene of interest was not replaced by the knockout cassette. If no rDNA amplicons are present, then the thermal shock procedure may need to be repeated. Also, this protocol has not been validated for successful PCR amplification from fungal hyphae, and, therefore, it is not guaranteed to work from such material.

In our hands, LongAmp Taq DNA polymerase works best with the primers and conditions tested. Phusion High‐Fidelity DNA polymerase also amplifies comparable PCR products (Fig. 6A); however, it was not extensively tested in this protocol. MyTaq RED Mix DNA polymerase proved to be the least efficient (Fig. 6B). If any other polymerases are to be used, we encourage users to validate them first.

The described protocol was optimized to work with the pathogenic fungus *A. fumigatus*, but it was also proven to be successful with *A. oryzae*, *A. flavus*, *A. nidulans*, and *Neurospora crassa*. No amplification was observed with *A. niger*; therefore, it is not a certainty that it works for any other fungal species. We recommend validating the above protocol if other fungal species are being used.

### Understanding Results

Basic Protocol 1 describes preparation of fungal spore suspension for PCR. For *A. fumigatus*, SAB agar is one of the most commonly used growth media to generate spores. The fungus is grown at 37°C for 24 to 72 hr and the generated spores are usually dark green in color for the laboratory wild‐type strains (spore color may vary for mutants or other strains). The spores are harvested in PBS–Tween 20 solution and used in Basic Protocol 2 for PCR. If other fungal species are being used, the user should optimize the growth conditions.

Following Basic Protocol 2, which describes PCR amplification from the spore suspension, the expected results are positive PCR bands observed on an agarose gel. As shown in Figure 4, this protocol works well for amplicons ranging between ∼600 bp and ∼5.3 kb; however, the recommended PCR amplification range is between ∼600 bp and ∼3 kb. If no PCR products are observed, refer to Critical Parameters and Troubleshooting.

### Time Considerations

The total time required to carry out this protocol depends on the number of fungal strains analyzed in parallel. Assuming that the fungal spores have already been harvested, the time required to obtain DNA from spores is less than 30 min. Subsequent PCR and gel electrophoresis take approximately 2.5 hr in total.

*A. fumigatus* laboratory strains (e.g., Af293) generate visible spores after approximately 1 day of incubation on SAB agar at 37°C; however, it is recommended to grow the fungus for an extra day to generate sufficient amount of spores before harvest. Other fungal species or genetically modified mutants may require different time for spore production.

Following incubation, spores are harvested into 15‐ or 50‐ml conical tubes in PBS–Tween 20 solution. This step takes no more than 5 min for a single fungal strain.

Setting up a PCR reaction for a single fungal strain requires no more than 10 min (may be longer for 96/384 plates), and the time of PCR will depend on the thermal cycler, the size of the amplified product, and the polymerase used. For a 1.5‐kb amplicon and the LongAmp Taq DNA polymerase, this step takes between 1.5 and 2 hr.
